# The impact of legal and organizational regulations on the nutritional behavior of patients-consumers of spa treatment services in Poland

**DOI:** 10.3389/fpubh.2022.1029505

**Published:** 2022-10-26

**Authors:** Joanna Wozniak-Holecka, Tomasz Holecki, Kajetan Suchecki, Sylwia Jaruga-Sękowska

**Affiliations:** ^1^Department of Health Promotion, Faculty of Health Science in Bytom, Medical University of Silesia in Katowice, Katowice, Poland; ^2^Department of Health Economics and Health Management, Faculty of Health Science in Bytom, Medical University of Silesia in Katowice, Katowice, Poland; ^3^Department of Market and Consumption, Faculty of Economics, University of Economics in Katowice, Katowice, Poland

**Keywords:** regulations, nutritional education, spa treatment facilities, health education, health care management, health economics

## Abstract

Health resort treatment facilities, regardless of the ownership status (public, private), most often carry out their activities based on contracts with the Polish public payer of the health care system, therefore the operation of sanatoriums is a fully-fledged element of the health care system, such as inpatient treatment, outpatient treatment and basic health care. The system designed in this way is designed to improve the organization of benefits, including by incorporating new useful organizational and legal tools. Thus, health education, along with its nutritional aspect, which is its inseparable part, is a perfect example of how to positively influence the behavior of consumers of spa treatment services. The structure of the study included the desk research method related to the consolidation and processing of information on applicable legal regulations and an individual in-depth, structured interview, using an original interview scenario. Based on the deliberate selection of the sample, 14 interviews were conducted. The interlocutors were representatives of the management of spa treatment facilities, academics and representatives of non-governmental organizations. The interview included functional and organizational, financial, competence and substantive aspects related to the implementation of nutritional education in spa treatment facilities in Poland. The use of a structured interview with experts made it possible to present the area of health education, including nutrition, in a broad light of the knowledge and experience of health care practitioners and theorists. Opinions were obtained on the optimal methods of assessing the effectiveness of education in the conditions of spa treatment and a set of system and organizational recommendations. Reporting of the results was presented using the COREQ checklist. It is justified to consider broadly understood health education in spa treatment facilities as a kind of wholesome health service, which may constitute effective systemic support for health policy and, consequently, increase the importance of prophylaxis and improve the health condition of the population. Such a legislative change will force organizational changes that may ultimately lead to a change in eating habits. Moreover, it is highly useful to use the potential of paramedical professionals, including dieticians, health educators and health promotion specialists.

## Introduction

Health should be treated as a dynamic, subjective, and difficult to measure concept, regardless of how one understands it, and attempts to define it are undertaken by various researchers (1, 2). According to the definition of the World Health Organization from 1947, it should be understood as “the complete, physical, mental and social well-being of a person, not just the absence of disease or ailment” (3). This definition strongly emphasizes the need to focus not only on the disease but also on health and its strengthening, taking into account the essence and role of prevention and health promotion. Public health experts point out that health also has an anthropocentric aspect and should be perceived as “the lack of a more serious disease and the individual's ability to function in a selected social environment and work environment leading to the achievement of life goals (4, 5)”. The presented idea aims to strive for a change toward the creation of a comprehensive model of patient-centered care, which allows improving both the health outcomes and the efficiency of the health care system in terms of the number of material resources, work, and financial resources necessary to achieve specific results. The system designed in this way is aimed at changing the relationship between services provided at different levels of the system and improving the quality of health care as a result of improving the organization of services, including by incorporating new useful tools, e.g., health and nutritional education within spa treatment.

Research on business models in relation to health tourism in the spa sector shows that the values created by the surveyed spa enterprises do not include direct benefits for the community, but serve only to achieve the economic goals of the enterprise resulting directly from delivering value to the client. It is widely believed that there is a need to broaden the concept of value proposition so that this component of the business model includes value for the customer, value captured by the enterprise, value for the community, and often also environmental benefits. The expected trend to create value for the community is predicted to be proportional to the effectiveness of the impact of customer value minus the value captured by the enterprise (6, 7). This is particularly important when, as is the case in Poland, spa treatment is an integral part of the health care system. Spa treatment is carried out in facilities located in areas with special geological and climatic values, designated for the purpose of using and protecting the natural healing resources there. Spa treatment establishments, regardless of the ownership form (public, private), most often carry out their activities based on contracts with the Polish public payer of health services (National Health Fund—NFZ) (8).

In 2019, the activity of spa treatment in Poland was carried out by 49 spa hospitals (including 6 for children), 192 spa sanatoriums (including 3 for children and 1 sanatorium in an underground mining excavation), 10 clinics and 20 natural medicine facilities servicing spa complexes. At the end of the year, hospitals and spa sanatoriums had 45.3 thousand places. During the year (2019), 782.1 thousand patients. This group was dominated by two subpopulations—women, who accounted for 60.8% of inpatient treatment, and people aged 65 and more (48.9%). The average stay of a patient in residential care was 16.1 days (9). In 2020, patient numbers fell by around 50% due to the COVID-19 pandemic (10, 11).

Unfortunately, despite the formal and organizational separation, no uniform regulations defining nutritional standards, methods of assessing the quality of nutrition, rules for employing dietitians, or average nutritional rates have been established, which significantly affects the quality of meals served in the surveyed medical entities (12, 13). Pursuant to the principles set out in the Act on healthcare services financed from public funds, patients have the right to healthcare services aimed at preserving, saving, restoring, or improving health and other activities resulting from the treatment process (14). Patients whose health condition requires full-day health services may be provided with other health services as part of, inter alia, spa treatment (15). According to 38 p. Article 5 of the above Act, food in a hospital or other treatment institution (e.g., a sanatorium) providing 24-h medical services in a stationary mode, should be adequate to the patient's health condition. Meals provided as part of the provision of services should not only ensure the 24-h energy and nutritional needs but also fulfill the function supporting the treatment process, which is of significant therapeutic importance (13). A properly balanced diet can shorten the time of treatment and convalescence, and thus reduce the costs of the entire process, for which the public payer pays. In 2011 Polish researchers cooperating with the Polish Food and Nutrition Institute indicated that this is a reduction of costs by up to 30–50% (16). The negative impact of improper nutritional status of patients on the effectiveness and costs of treatment is reflected in the increased frequency of complications of existing diseases, worse treatment outcomes, and extended hospitalization time (17). As a consequence, this causes an increase in the healthcare system payer's expenses for services financed from public funds, as well as expenses incurred by consumers on a commercial basis.

In accordance with the principles of proper nutrition, it is necessary to assess the nutritional status of patients at the time of their arrival for spa treatment, in order to determine the nutritional needs resulting from the diagnosed disease entities (16). In order to give proper importance to the nutrition issues in Poland, it is necessary to design modern systematic solutions. Moreover, the development of standards and requirements for nutrition in spa treatment facilities will avoid the common lack of rules regarding the energy and nutritional value of the meals served. This is despite the valid at the time of the research recommendations of the Nutrition and Food Institute (in 2020 National Institute of Public Health—National Institute of Hygiene) in the field of diet nomenclature. There are known cases of using different, outdated names of diets in a spa treatment facility, resulting in assigning wrong goals and implications to them. What is more, the method of nutrition of the patient during the treatment stay was largely decided by a medical doctor, less often a dietitian, but there are also some disturbing practices of the patients themselves choosing their diets (18). Reports of the Polish Supreme Audit Office from 2008−2018 (19, 20) showed numerous irregularities in hospital nutrition, which may raise doubts as to the quality of nutrition in other medical facilities, which include, among others, sanatoriums.

In order to unify and simplify the regulations on nutrition in medical institutions, it would be necessary to specify the requirements and standards for proper nutrition of patients, specifying the minimum nutritional rate, as well as the obligation to employ a nutritionist who would take care of the patient and supervise the implementation of the established nutritional plan. Incorrect nutrition in non-sanatorium conditions may have a negative impact on health or lead to the aggravation of the disease, and as a consequence, the effects obtained during spa treatment will be weakened or completely disappear (16). Therefore, an inherent element of spa treatment is initiating, supporting, and monitoring activities in the field of nutritional education aimed at raising awareness in this regard, and, if necessary, directing to change the existing eating habits. Services that should be provided to a patient referred to spa treatment or rehabilitation are described in the basic legal act for the industry, i.e., the Act on spa treatment (21) and the Regulation of the Minister of Health on guaranteed benefits in the field of spa treatment (22). These acts and regulations clearly emphasize the role of health education, nutritional education, and ensuring the correct diet of patients treated in the sanatorium. Legislative strengthening of this legal act by issuing executive acts should be considered a priority, because nutritional education, as an important element of health education in the prevention of food-related diseases, may reduce the incidence of cardiovascular diseases, overweight and obesity, as well as diabetes, gastrointestinal diseases, and osteoporosis, which are the most common reasons for referring a patient to a spa treatment.

The aim of the research was to find the determinants of health education implemented in spa treatment facilities in Poland, in the context of legal and organizational processes, and the final result of the study was the outlining of recommendations for changes in this area.

## Materials and methods

The data used in this study was collected through qualitative research. This type of research is burdened with a certain methodological imprecision, due to the fact that different social sciences researchers give their understanding of qualitative research differently, and they question the need to prepare an interview scenario (23). However, research using the in-depth interview method (IDI) makes it possible to describe reality not with the help of numbers, which do not always allow for the correct interpretation of data, but use words, which not only allows for the presentation of certain facts, but also attempts to provide reasons and connections between various phenomena (24).

In the case of qualitative research, researchers initially tried to adopt the classic criteria of empirical social endowments, i.e., reliability, validity and objectivity. It turns out, however, that this reliability, traditionally understood as the possibility of a stable nature of data and the results of subsequent research, is practically impossible to implement. The criterion of validity is also contested. Relevance is optimized by means of far-reaching standardization, which, from the point of view of the nature of qualitative research, is extremely difficult, and sometimes even contradicts the idea of this research. The objectivity of a direct interview should be considered in four respects: freedom from prejudices, intersubjective consent, relevance to the subject of the study, and the interviewee's ability to “resist” the researcher (25).

There is a great variety of qualitative data. Among them, we can distinguish: individual and focus interviews with their transcriptions, e-mails, websites, participant observation, video journals, diaries, photos and films (26).

Based on the methodological assumptions, the study was conducted in 2016–2018 using qualitative methods—IDI (Individual In-Depth Interviews). An interview scenario was used for the better quality of the results obtained during the study. This scenario was divided into three main parts, corresponding to the topic of the interview. The scenario is attached as an annex.

For the proper conduct and reporting of this research, the COREQ Checklist (COnsolidated criteria for Reporting Qualitative research) was used, in which 32 elements necessary for the proper presentation of the methodology and results of qualitative research were noted (27). This form is attached as an annex.

The research consisted in conducting a structured interview, the effects of which allowed to obtain specific information in a planned and methodical manner, according to an author's scenario. The standardization of the issues raised, and of the purpose of the interview, in particular, contributed to obtaining information relevant to the objectives of the study. Moreover, it enhanced the understanding of the issues raised and the patterns revealed. The number of managers was equal to the number of scientists. People dealing with the subject of health resorts from the scientific point of view came from leading university centers in Poland, dealing with health policy, health promotion, health education, public management, health tourism and management in health care. Each spa was represented by one manager, with the exception of one facility where the interview was conducted in the form of a dyad conducted with two experts representing one center.

The research was direct—face to face. The interviews were conducted by a substantively prepared female researcher, assistant professor at the university, co-author of this work (JW-H), with 20 years of experience in conducting scientific research and holding a doctoral degree in health science, having scientific achievements in the field of the problem in question and implementing two research projects on the issues of organizing health education in spa treatment facilities, using a dictaphone, and then the obtained answers were rewritten. During the interviews, the researcher also took notes, writing down the most important elements of the conversation. Due to the experience and knowledge of the person conducting the research (scientist, who is also a co-author of the study), guides and guides for interviewers were not used. Each interview lasted approximately 60 min. The selection of the sample was purposeful, 14 interviews were conducted, 2 of which were in the form of dyads, which means the researcher interviewed two people at the same time. It is acceptable from a methodological point of view (28, 29). The interviews were conducted with representatives of the management of spa treatment facilities, academics with scientific achievements in the field in question, and representatives of non-governmental organizations dealing with senior issues. Due to the fact that some of the respondents came from the university environment, some of the respondents were known to the researcher. They were informed that the research results would be used for scientific purposes. The interview form has been divided into parts related to: functional and organizational, financial, competence, and substantive aspects related to the implementation of health and nutrition education in spa treatment facilities in Poland.

The research was data-gathering in order to analyze the content of experts' statements. There were also no re-interviews. There was also no discussion of data saturation, and no transcripts were sent to participants for corrections or comments. There was also no feedback from the participants.

The person conducting the research was also the only coder of the results, and the topics about which the discussions were conducted were identified before starting the research. Due to the nature of the research, no advanced computer software was used, but only a Microsoft WORD text editor to prepare a transcript of the statements of the study participants. For a better and more understandable reception of the results, the quoted statements are not exact quotations. They also often express the opinion of more than one person. The presented results refer directly to the analysis of data obtained in the study based on the literature on the subject. The results focus on the presentation and analysis of data corresponding to the research topic without going into the details of the so-called minor topics raised by the respondents during the interview.

## Results

### Legal regulations

In view of the issues of health education and nutritional education and their tools integrated into the spa treatment system in Poland, the experts drew attention to the heterogeneity of the recipient group—consumers of spa treatment services, for whom adequate educational tools should be addressed. In most cases, it was declared that the integration of health promotion and related activities (including nutritional education) into the treatment process is justified, the more so that the issues of health promotion, health education, and disease prevention actually implemented in the health care system are firmly embedded in the structure of the treatment process and organizational tradition of the system. In the case of considerations about the possible recognition of health education as a guaranteed service, and therefore controlled and evaluated by the entity paying for its implementation, there was a discrepancy between the representatives of theoretical thought and the practices organizing the services. The scientists' assessment was critical, as they believed that these types of instruments are only an auxiliary form and may be an obstacle to achieving real benefits from a stay in a spa facility, which is due to the fact that the stay is to be not only medical but also recreational. On the other hand, the representatives of the organizers expressed their conviction that the actions aimed at expanding educational activities were right, referring to the experiences of highly developed countries and paying attention to the necessity to obtain financial strengthening of the undertaken activities.

### Legal and organizational regulations

Conducting a separate contracting, reporting, or targeted control solely in the educational process was accepted in the opinions of experts, although the technical and organizational difficulties of such solutions were unanimously emphasized. However, in the face of under-financing and the lack of solutions allowing to control the rationality of the expenditures, a problem may arise in the form of institutions striving to maximize their own income at the expense of the patient. In the financial dimension, we are then dealing with a deficit of resources, with the simultaneous lack of mechanisms forcing their proper allocation. Perhaps a solution could be the use of lump-sum settlements supplemented with the FFS (Fee For Service) method and/or payment for results using the P4P (Pay For Performance) method.

In practice, despite the statutory provisions on the obligation to provide health education in spa treatment facilities, some service providers do not comply with these provisions. According to experts, underestimating the implementation of health and nutritional education in health resorts results from the fact that patients do not know that they may require this service from the facility and the fact that they often treat their stay as leisure rather than an opportunity to learn and change their eating habits. The above two reasons are combined with the problem of the lack of monitoring of educational activities, poor control or supervision process, not to mention measuring their quality or the degree of exerting an influence on the future behavior of recipients. In the opinion of the majority of respondents, putting the right attention to the implementation of the contracted, directly or partially, procedures should be considered one of the most important tasks in the context of maintaining or strengthening the role of the educational procedure in spa treatment. Among many expert opinions, there were always opinions that the elements of education could not be excluded from health promotion activities provided by spa treatment entities, and very often they were even considered the foundation for further effective activities in working with patients. In their opinion, health education is an important element determining the complexity of the service, and the stay at the spa is not only aimed at improving health but is also carried out in order to learn about certain habits harmful to health (with particular emphasis on eating habits) and develop practices related to strengthening healthy behavior. In fact, among the specialists interviewed, the complexity of health services supplemented with the elements of education has always been recognized as an asset that should be pursued. Experts pointed out that the service would be comprehensive only if the acquisition of specific knowledge, e.g., nutritional knowledge, goes hand in hand, for example, with a diet appropriate to the age and health of the patient. In the talks on the complexity of services, there were also voices critical of the current role ascribed to educational activities, especially of their inadequate financing and implementation methods.

A very important area of the study was to determine whether it is necessary to additionally sanction in the legislative process a list of persons authorized to conduct health education in spa treatment entities through their profession, competencies, requirements, courses, or training. In principle, there is no reason to deny the usefulness and competence in the field of health education of the current professional groups permanently associated with medicine, such as medical doctors, nurses, or nutritionists, and their involvement should be completely natural and desirable. It can also be assumed that if a new service is satisfactorily priced, there will be people willing to expand their qualifications in the area of professional nutritional education, especially in the group of the secondary medical personnel. However, special attention should be paid to the catalog of necessary competencies for people outside the core of the medical profession. The new profession of health educator must naturally contain provisions conditioning the possibility of training in this area and should go hand in hand with legislation, both, in the statutory and supplementary scope in the form of executive and regulatory acts. The organization of the profession of an educator in health care requires professional preparation of staff, their location, and systemic structuring, as well as ensuring a process of continuous improvement of skills.

### Organizational regulations (including management and financial regulations)

In view of the coordination processes, as well as the wide view of the patient's functioning in the Polish health care system, all respondents expressed their opinions very uniformly. In their opinion, education in spa treatment facilities should be based on a developed system of standards, the same for all payers. Establishing a specialized control agency or thematic units in existing institutions was also deemed unnecessary. In terms of shaping eating habits, special attention was paid to the coordination of processes in which the patient should benefit from continuous health education, regardless of the type of health care facility, as well as the stationary or environmental model. It was emphasized that in a patient undergoing a stay in the subject of spa treatment, the habits developed under the health care should be consolidated, and behaviors that should be transferred to the home environment should be shaped in the spa. This process should be supervised by the educator in both of the above-mentioned areas of influence, who would consolidate these habits.

According to experts, without introducing a sufficient price for the offered product, it is difficult to expect the involvement of staff in its implementation, which, moreover, should be subject to control in terms of mileage and, consequently, high efficiency. It was agreed that health education is a process that should be monitored and then evaluated.

Assuming that, as most experts recommend, health education should become a guaranteed service, financed by a public payer, the capitation rate, i.e., linking the valuation with the number of people covered by the service, was indicated as an adequate financing model. However, other forms of valuation and billing were also not excluded, taking into account the simplest accounting forms described by the respondents as the hourly rate of the educator's work or the equivalent for man-hour of education. The problem of additional administrative workload and an increase in the number of settlement formalities arising from such a financing model, resulting in inevitable financial and organizational costs was also noticed.

The aspect of selecting professional health educators was considered extremely important. Experts agree that the current HR model used in sanatorium health education is ineffective and requires a radical qualitative change. Conducting health education by medical doctors, nurses or physiotherapists was considered common, but misplaced and unequivocally wrong. As a target, it was recommended to introduce a qualified staff of professionals representing medical-related professions, including nutritionists, public health specialists, personal trainers, who will have a range of competencies adequate to the tasks related to health and nutrition education, into the system. The optimal solution would be to distinguish a separate category of service and a separate category of a specialist who will provide it professionally. The respondents expressed the opinion that the above-mentioned educators should constitute the foundation of the undertaken activities, which, however, will be implemented in cooperation with other staff of spa entities. The justification for this type of solution is also the pragmatism resulting from the deficit in the market of typically medical professions and the disproportion of the rates offered to them. The possibility of using the latest techniques of influencing the behavior of people undergoing training was also noticed, however, due to the high costs of their implementation and application, it is currently considered only theoretical.

In the part on financial and organizational effectiveness, experts pointed out that it is very difficult to refer to comparable studies from highly developed countries or countries undergoing a systemic transformation so as to demonstrate the effectiveness or ineffectiveness of such solutions on the basis of Evidence-Based Policy. The value of the positive consequences of implementing new solutions on a national scale was also emphasized and such a perspective was recommended.

In detail, the issues of technical solutions related to the implementation of training were also discussed. Some experts clearly recognized the advantage of group education over individual education, justifying it with the tasks of the state and the consequence of preparing actions tailored to epidemiological needs, taking into account selected disease entities, as well as cost optimization. The others argued completely differently, giving a clear primacy of the individual activities over the group ones, resulting from the effectiveness of educational processes. This type of perception of the discussed issues resulted from the belief that selected groups of people struggling with similar problems would mutually reinforce each other. In this case, the justification is based on work in small groups of exercises or workshops.

### Presentation of the results in aggregate form

For a clearer presentation of the above opinions, experts' observations and the consequences of these identified challenges, they were grouped into three categories and presented in the Tables: in the scope of legal regulations (Table 1), in the legal and organizational scope (Table 2) and in the organizational (including management and financial) scope (Table 3).

**Table 1:** Opinions and observations of experts in the field of legal regulations.

**Opinions and observations of experts**	**Possible consequences or ideas for the future**
Inadequate ways of financing and implementing educational activities according to the law.	Poor quality of health education services, or even giving up such activities.
Separating health and nutrition education for settlements with the public payer is the right solution	Increasing the number and improving the quality of services in the field of health and nutrition education.
Recognition of health and nutrition education as a guaranteed benefit.	Possibility of financing by a public payer. Proposals of the *per capita* rate, but also other forms of calculation. Risk of additional administrative costs.
Legal sanctioning of the list of persons authorized to conduct health and nutritional education.	It may be easier to obtain properly prepared and educated staff in the field of health and nutrition education.

Own study based on qualitative research.

**Table 2:** Opinions and observations of experts in the field of legal-organizational aspects.

**Opinions and observations of experts**	**Possible consequences or ideas for the future**
Necessity to formalize educational issues.	Formalization should be a management aid, not an obligation.
Separate financing and contracting of health and nutrition education should be introduced.	It is an opportunity to improve the quality of health and nutrition education services.
Coordination of education in sanatoriums should be substantive, not organizational.	Educational processes should be supervised by educators, including dieticians.
The control of the educational process improves its efficiency.	It is necessary to monitor the process and then evaluate it.

Own study based on qualitative research.

**Table 3:** Opinions and observations of experts in the field of organizational tasks (including management and financial aspects).

**Opinions and observations of experts**	**Possible consequences or ideas for the future**
We can talk about the heterogeneity of patients - consumers of spa treatment services	Educational tools appropriate to the type of consumer should be used. However, this results in increased costs and management difficulties.
In sanatoriums, the legal acts concerning the education of patients are being downplayed.	Need for more comprehensive service delivery audits.
Patients' lack of knowledge about the right to health and nutrition education.	Some people who would be willing to receive health and nutrition education may not take advantage of it due to their ignorance.
A stay in a sanatorium should be comprehensive, holistic, and therefore also educational.	The necessity to transfer knowledge and competences in the field of health promotion and proper nutrition.
There is no specific price for the service—health and nutrition education.	Lack of staff involvement in the implementation of these tasks
Ineffective selection of educators—mainly medical doctors, nurses and physical therapists.	It is worth using the knowledge of dietitians, public health specialists or even personal trainers. It will also result in lower costs of health education services.
There are no studies in this area from the so-called Western countries.	Problems relating to financial and organizational effectiveness.
Activities in the field of spa treatment should refer to the general (global) evaluation.	Striving to improve the health of the population and avoiding health risks.
The technical and organizational implementation of trainings is an extremely important issue from the point of view of the effectiveness of the entire health education process.	Some experts opt for individual training. Others prefer group ones. This should depend on the one hand on cost optimization and on the other hand on research on the effectiveness of the educational process.

Own study based on qualitative research.

## Discussion

Health education in spa treatment entities is a basic tool for health promotion, including the promotion of proper eating behavior, enabling the introduction of a permanent change in the lifestyle of patients. In the light of the law (17), this type of therapy can be treated as a repetitive procedure. Cyclicality is also a basic condition for the effectiveness of the health education process because only regular and systematic activities in this area can strengthen the knowledge and health habits of its participants. For this reason, spa treatment facilities seem to be an excellent place to carry out tasks in the field of patient education. For this reason, spa treatment facilities seem to be an excellent place to carry out tasks in the field of patient education, but also local communities. Research shows (7, 30) that spa entities often undertake initiatives supporting the local community, especially in the field of education, health, sanitary safety and mitigating the negative effects of climate change.

However, in order to be able to talk about sanatorium education as an element of coordinated patient care, it would be necessary to undertake systemic actions favoring, and in a way forcing, the continuity and complexity of this care (31). The optimal element of coordination, located in the Polish health care system, is the Primary Healthcare, which employs medical doctors who are closest to the patients and know their specific health needs. Thus, doctors employed in Primary Health care clinics should receive feedback from the spa treatment system, including information on the educational training received and its final effect related to a possible change in behavior patterns. Unfortunately, comparative studies show that this level of healthcare provision also requires a major overhaul. Poland fares worse than other European countries in terms of organizational governance, an example of which is e.g., problems with financing, the constant development of human resources, and implementation plans, including, inter alia, educational training. Although Primary Healthcare plays a major role in the delivery of commonly available services, no evaluation processes and tools for monitoring, assessing, increasing, and consolidating the effectiveness of the services provided have been developed (32). Parallel strengthening and coordination activities are absolutely necessary in the presented situation, because the seemingly independent and dispersed between the interpenetrating organizational layers of the Polish health care system, elements such as activities in the field of education in spa treatment, consists of the final health result achieved on a population scale. Attempts to rationally shape its development require systematic recognition of these conditions. The effectiveness of training depends, on the one hand, on the support of the health and social policy, there is a need for legislative changes and the creation of an education system based on the letter of the law within the spa treatment. Another key element is the attitude of healthcare professionals to the importance and importance of such activities. Recognizing health education as a type of service to which specific procedures will be assigned and which will be rationally priced, could undoubtedly raise the importance of training. The third important component of effectiveness is shaping appropriate attitudes and ways of thinking of the general public (recipients of messages) about the importance of health education in their everyday life. It is necessary to consolidate the common belief that health promotion, including nutritional education, should become an element of the social system of values.

One of the ways of implementing health education in spa treatment is the idea of developing training cycles compatible with the individual treatment directions of sanatoriums. Conducting health education, nutritional education, and health promotion directed in accordance with the therapeutic profile, extended with a specific therapeutic diet adequate to the patient's disease, is also listed in the list of guaranteed services in the field of spa treatment (33, 34).

Attention should also be paid to the unused potential of public health specialists in supporting medical staff in the processes of transferring knowledge to patients. The method of disseminating health education based on the profession of educator opens up new opportunities for increasing both the standard and the scope of activities in the area of health promotion and prevention, which enables quality improvement at all levels of the health care system. Strengthening health promotion and prevention can help to slow down huge cost increases in this sector. The directions of activities so far focused on the implementation of these activities by medical professionals for whom conducting health education is only one of numerous, yet by no means the primary, professional tasks. Medical personnel will always put disease treatment in the first place among their goals, which is obvious, but at the same time, it is associated with relegating health promotion activities to the background. Therefore, it is recommended to employ non-medics, but only medical-related persons as a therapy coordinator who is to accompany the patient during the entire stay at the spa, e.g., public health specialists.

On the other hand, health educators could develop and use at work the knowledge and skills characteristic of their profession, as well as significantly support the treatment processes, providing, in cooperation with medical doctors and other medical professionals, such as nutritionists, extensive information support concerning, above all, further treatment and rehabilitation but also including information of an organizational nature (35). In the literature on the subject, there is a suggestion to make the educator a patient's guardian, as it is commonly called, or even a therapy coordinator, who would accompany the patient during the entire stay at the spa, taking care of health education, also planning treatments and contacting other specialists employed in the facility. The presented solutions are in line with the global tendency to delegate some tasks that do not require highly specialized medical competencies to other specialists who are not doctors, e.g., prescribing certain medications by nurses or the analysis of some tests by medical technicians. In management sciences, such solutions are referred to as “skillmix.” This enables a more effective allocation of human resources and, at the same time, brings considerable savings (36).

In order to determine the development needs, it is necessary to analyze the existing state of competencies and define the target state. Educational goals should be clear to both trainers and participants, achievable, and easy to measure. It should also be noted that in order for the training to be effective, it must be constantly updated in the future. Each subsequent implementation is associated with its improvement and modification so that it optimally meets the needs of the participants. All decisions related to the type of examination performed, the method of treatment, or the scope and necessity of the services provided, are now made by medical personnel, because it is difficult to expect a wider assessment and selection of a health service from the patient, due to the significant asymmetry of information (37). As indicated by the international research achievements to date, most patients do not have sufficient expert knowledge and skills to assess whether the provided health service was necessary and whether it was performed correctly (38). The optimal solution would, of course, be the implementation of nutrition science in the education process at every level of education, but until this happens, it should be assumed that the education process in the health resort will focus primarily on eliminating the disproportionate knowledge between patients. Currently, due to objective reasons, i.e., the lack of uniform assessment criteria, institutions financing spa treatments often only take into account the opinion of patients about the price of services (often in isolation from the market quality) and the material standard of facilities (39). On the other hand, the introduction of the quality of services provided to the patient's measurement system is justified, because the patient is the beneficiary of the treatment process. In addition, the patient experiences direct contact with medical staff and is a user of all kinds of devices related to the process of providing health services and as such is an excellent source of information used to verify the spa therapy process (40).

A training project should be assessed primarily in terms of its effectiveness. Without such an assessment, it is not possible to state whether the adopted goals have been achieved, and then whether the participants have developed their competencies to a satisfactory degree. Measurement of the effectiveness of the training project also provides information on whether the transfer of new knowledge has been made and whether the expected changes have occurred as a result of it, having a positive impact on the improvement of effectiveness. The evaluation of a training project is a summing-up stage that gives guidance in the human resource development planning process and is a very important, but still underrated, element of the training project. Since each activity involves a certain financial outlay, measuring the effectiveness of training projects is also a natural need to verify that the investment in human resources was profitable (41). In the case of spa treatment, the quality control mechanisms of the educational process implemented by some payers contribute to a higher level of patients' satisfaction with these activities.

## Conclusion

Although the opinions of experts were not always consistent, the most important problems in the discussed area included those related to the financing of health education—as part of a larger service, and thus often neglected. Staffing problems are also important—there is a shortage of educators or nutritionists working in spa treatment facilities, and their role is often taken over by doctors or nurses. These people, despite their high competences, usually treat these duties as additional or even irrelevant. Moreover, doctors' labor costs are relatively high, and the use of educators would allow them to be reduced in the health care system. It is difficult to require the managers of institutions to treat health education services seriously, if the systemic and legal solutions do not make it easier. Proper implementation of education, from the point of view of the company's interests, may generate additional costs with no (or little) additional income. These changes are not possible without organizational and legal changes in the Polish health care system.

The system requires the implementation of a set of coordinated legal provisions in order to have a positive impact of the regulation on the functional and organizational change in spa treatment facilities in terms of shaping the nutritional behavior of patients.

Recognition of health education, including nutrition education, in spa treatment facilities as a kind of wholesome health service, analogous to other health services financed by a public payer, may constitute effective systemic support for health policy, including the senior policy, in connection with the demographic changes currently taking place in the Polish population. Although state expenditure on spa treatment does not exceed 1% of the budget of the National Health Fund (42), and most of the units providing treatment are small private companies, due to the importance of the solutions described, ready proposals for financing these services should lie with the health care system.

Education in spa treatment should be a continuous, structured, and standardized process within the entire system so that it can be monitored and evaluated on an ongoing basis based on standardized effectiveness measures.

The implementation of proper health and nutritional education in spa treatment facilities should aim at improving patients' understanding of the role of nutrition in their health, in particular after their stay in the facility. It would be worthwhile to conduct direct research among patients who have used these types of health promotion services, whether there have been any long-term changes in their diet and general health after leaving the centers. This type of study would complement the present study, which was largely normative.

It should be recognized that the conclusions of this study may be useful, in particular, for those managing the health care system in Poland but also in other countries with a similar model of the health care system, including the EU and EFTA countries.

Among the limitations resulting from the conducted research, it is worth mentioning the limited group of experts participating in the research. The perspective of further research should include further descriptive and comparative analyzes on a larger sample of respondents, taking into account the perspective of spa stakeholders and patients. It is also worth conducting a similar post-Covid study to determine how the situation in the study area has changed.

## Data availability statement

The original contributions presented in the study are included in the article/supplementary material, further inquiries can be directed to the corresponding author.

## Author contributions

Conceptualization, investigation, data curation, writing-original draft preparation, supervision, project administration, and funding acquisition: JW-H and SJ-S. Methodology: JW-H, KS, SJ-S, and TH. Validation and formal analysis: TH and KS. Writing-review and editing and visualization: JW-H, SJ-S, TH, and KS. All authors have read and agreed to the published version of the manuscript.

## Funding

The research was financed by the Ministry of Science and Higher Education of the Republic of Poland granted as part of statutory research carried out at the Faculty of Health Sciences in Bytom, Medical University of Silesia in Katowice, numbers KNW-1-049/N/6/Z and KNW-1-056/K/7/Z.

## Conflict of interest

The authors declare that the research was conducted in the absence of any commercial or financial relationships that could be construed as a potential conflict of interest.

## Publisher's note

All claims expressed in this article are solely those of the authors and do not necessarily represent those of their affiliated organizations, or those of the publisher, the editors and the reviewers. Any product that may be evaluated in this article, or claim that may be made by its manufacturer, is not guaranteed or endorsed by the publisher.
